# Sexually dimorphic methylation patterns characterize the placenta and blood from extremely preterm newborns

**DOI:** 10.1186/s12915-023-01662-7

**Published:** 2023-08-23

**Authors:** Hudson P. Santos, Adam E. Enggasser, Jeliyah Clark, Kyle Roell, Vasyl Zhabotynsky, William Adam Gower, Diana Yanni, Nou Gao Yang, Lisa Washburn, Semsa Gogcu, Carmen J. Marsit, Karl Kuban, T. Michael O’Shea, Rebecca C. Fry

**Affiliations:** 1 School of Nursing and Health Studies, University of Miami, Coral Gables, FL USA; 2 Gillings School of Global Public Health, Institute for Environmental Health Solutions, University of North Carolina at Chapel Hill, Chapel Hill, NC USA; 3 Department of Environmental Sciences and Engineering, Gillings School of Global Public Health, University of North Carolina at Chapel Hill, Chapel Hill, NC USA; 4 Department of Pediatrics, School of Medicine, University of North Carolina, Chapel Hill, NC USA; 5 Department of Neonatology, Beth Israel Deaconess Medical Center, Harvard Medical School, Boston, MA USA; 6 Department of Pediatrics, Wake Forest School of Medicine, Winston-Salem, NC USA; 7 Department of Environmental Health, Rollins School of Public Health, Emory University, Atlanta, GA USA; 8 Division of Pediatric Neurology, Department of Pediatrics, School of Medicine, Boston. University, Boston, MA USA; 9 Curriculum in Toxicology and Environmental Medicine, University of North Carolina at Chapel Hill, Chapel Hill, NC USA

**Keywords:** Epigenetics, Placenta, Blood, Preterm birth, Child health

## Abstract

**Background:**

Health outcomes among children born prematurely are known to be sexually dimorphic, with male infants often more affected, yet the mechanism behind this observation is not clear. CpG methylation levels in the placenta and blood also differ by sex and are associated with adverse health outcomes. We contrasted CpG methylation levels in the placenta and neonatal blood (*n* = 358) from the Extremely Low Gestational Age Newborn (ELGAN) cohort based on the EPIC array, which assays over 850,000 CpG sites across the epigenome. Sex-specific epigenome-wide association analyses were conducted for the placenta and neonatal blood samples independently, and the results were compared to determine tissue-specific differences between the methylation patterns in males and females. All models were adjusted for cell type heterogeneity. Enrichment pathway analysis was performed to identify the biological functions of genes related to the sexually dimorphic CpG sites.

**Results:**

Approximately 11,500 CpG sites were differentially methylated in relation to sex. Of these, 5949 were placenta-specific and 5361 were blood-specific, with only 233 CpG sites overlapping in both tissues. For placenta-specific CpG sites, 90% were hypermethylated in males. For blood-specific CpG sites, 95% were hypermethylated in females. In the placenta, keratinocyte differentiation biological pathways were enriched among the differentially methylated genes. No enrichment pathways were observed for blood.

**Conclusions:**

Distinct methylation patterns were observed between male and female children born extremely premature, and keratinocyte differentiation pathways were enriched in the placenta. These findings provide new insights into the epigenetic mechanisms underlying sexually dimorphic health outcomes among extremely premature infants.

**Supplementary Information:**

The online version contains supplementary material available at 10.1186/s12915-023-01662-7.

## Background

Individuals born extremely preterm are at increased risk of adverse neonatal and developmental outcomes including sepsis, necrotizing enterocolitis, respiratory distress, cerebral palsy, cognitive impairment, epilepsy, autism spectrum disorder (ASD), and attention deficit hyperactivity disorder (ADHD) [1, 2]. However, the likelihood of these outcomes is not equal for males and females [3]. In general, males are at higher risk for detrimental health outcomes such as ADHD, ASD, and a plethora of other morbidities compared to females [1, 4–7]. Although these sexually dimorphic outcomes are well-documented, the underlying mechanisms are understudied.

One important molecular mechanism that may influence sexually dimorphic health outcomes is epigenetic DNA modification through CpG methylation. CpG methylation represents the addition of methyl groups to cytosines that can result in gene suppression or activation without changing the nucleotide sequence [8]. CpG methylation contributes to the regulation of important biological processes during early life development such as transcription, genomic imprinting, X-chromosome inactivation, and pluripotency [8, 9]. Males and females are known to have different CpG methylation patterns across the genome and thus is a potential mechanism underlying the male disadvantage in developmental outcomes [10–17]. This sexual dimorphism has been observed in the placenta, cord blood, umbilical artery, and brain tissue methylation levels across the lifespan, demonstrating the breadth and stability of dimorphisms [10–17]. Though not clearly understood is its relation to early developmental outcomes, sex differences in methylation patterns in target tissues might underlie, at least in part, sexual divergences in health outcomes [15, 16].

Although the methylation levels of some CpG loci may display conservation across tissues, methylation patterns significantly vary between tissues and are an important part of cell differentiation [18]. Thus, one tissue sample cannot provide a comprehensive assessment of sexually dimorphic molecular changes associated with developmental outcomes. A comparison of the epigenomes within the placenta and blood is warranted, as their CpG methylation signatures have both independently been linked with prenatal exposures, developmental outcomes, and sexual dimorphisms [12, 15, 18–30]. The placenta has been studied as a sensor and conductor between the mother and the fetus, playing an important role in fetal tissue growth, vascularization, and hormone production [31]. Additionally, it facilitates the supply of nutrients from mother to child and filters harmful substances to protect the fetus [31]. Placental CpG methylation has been associated with both short- and long-term outcomes of extremely premature newborns such as cognitive and socio-behavioral impairment, increased body mass index (BMI), and retinopathy of prematurity [19, 32–35]. Methylation differences between male and female placentas have been observed, with male placentas typically hypermethylated in males [15]. Similarly, CpG methylation from neonatal blood has been associated with clinical outcomes such as ASD, measures of BMI, and insulin sensitivity [24–26]. Unlike the placenta, cord blood tissue is typically hypermethylated in females [10, 11].

While CpG methylation in placental tissue and blood has been studied independently, sexually dimorphic patterns in these two tissues have not been compared. In the present study, we aim to characterize and compare sexually dimorphic DNA methylation patterns in the placenta and neonatal blood on day 1 in extremely premature newborns. Specifically, this study will determine the extent of sexually dimorphic methylation in the placenta and neonatal blood, contrast average methylation levels between male and female newborns, and examine biological pathways associated with differentially methylated genes. We hypothesize that most sexually dimorphic CpG sites will be tissue specific.

## Results

### Study subject characteristics

The general characteristics of study participants (*n* = 358) for the present study are described in Table 1. Most mothers were between the ages of 21 and 35 years and completed high school education or more (86.3%) and did not smoke (87.7%). The mean gestational age was 26 weeks, ranging from 23 to 27 weeks. The mean birth weight was 828 g, ranging from 420 to 1420 g. In total, 190 (53.1%) males and 168 (46.9%) females were included in this analysis.

**Table 1:** Demographic data for subjects for the current study (*n* = 358). Distributions summarized as the mean [min–max] or *n* (%)

	**Overall (*n* = 358)**	**Females (*n* = 168)**	**Males (*n* = 190)**
Maternal age (years)	29 [14–44]	28 [15–44]	29 [14–44]
Maternal education			
< 12 years	49 (13.7)	19 (11.3)	30 (15.8)
12 to 16 years	165 (46.1)	82 (48.8)	83 (43.7)
16 + years	134 (37.4)	64 (38.1)	70 (36.8)
Missing	10 (2.8)	3 (1.8)	7 (3.7)
Smoking during pregnancy			
No	314 (87.7)	146 (86.9)	168 (88.4)
Yes	38 (10.6)	20 (11.9)	18 (9.5)
Missing	6 (1.7)	2 (1.2)	4 (2.1)
Gestational age (weeks)	26 [23–27]	26 [23–27]	26 [23–27]
Birth weight (g)	828 [420–1420]	811 [430–1360]	843 [420–1420]

### Sexual dimorphism of DNA methylation across the placenta and neonatal blood

**Fig. 1:**
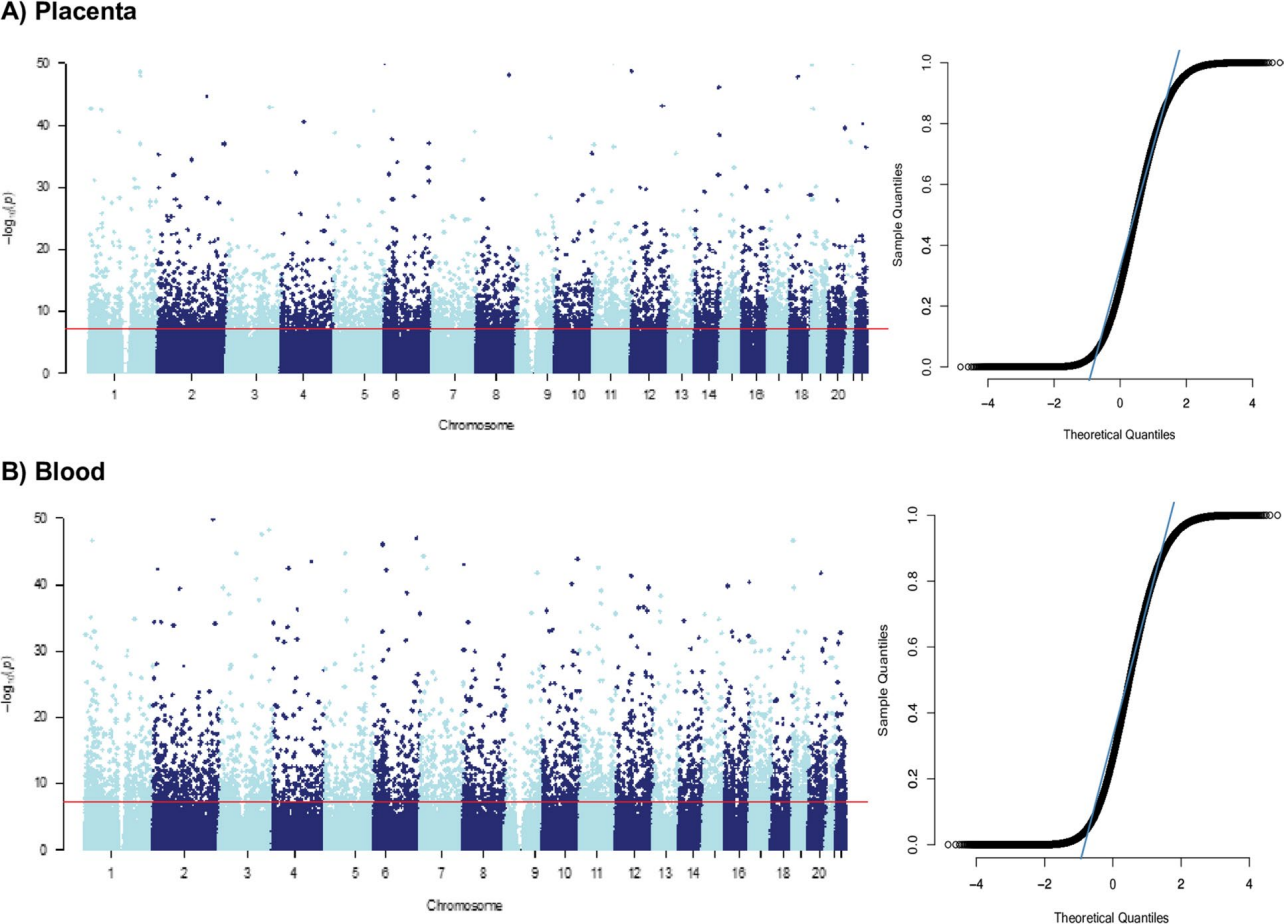
The left side shows Manhattan plots with the distribution of autosomal sexually dimorphic CpG sites in (**A**) placenta (*n* = 6182) and (**B**) day 1 blood (*n* = 5594). The right side shows the QQ plots visualizing displaying the genomic inflation for placenta (*λ*gc = 2.74) and blood (*λ*gc = 2.76)

**Fig. 2:**
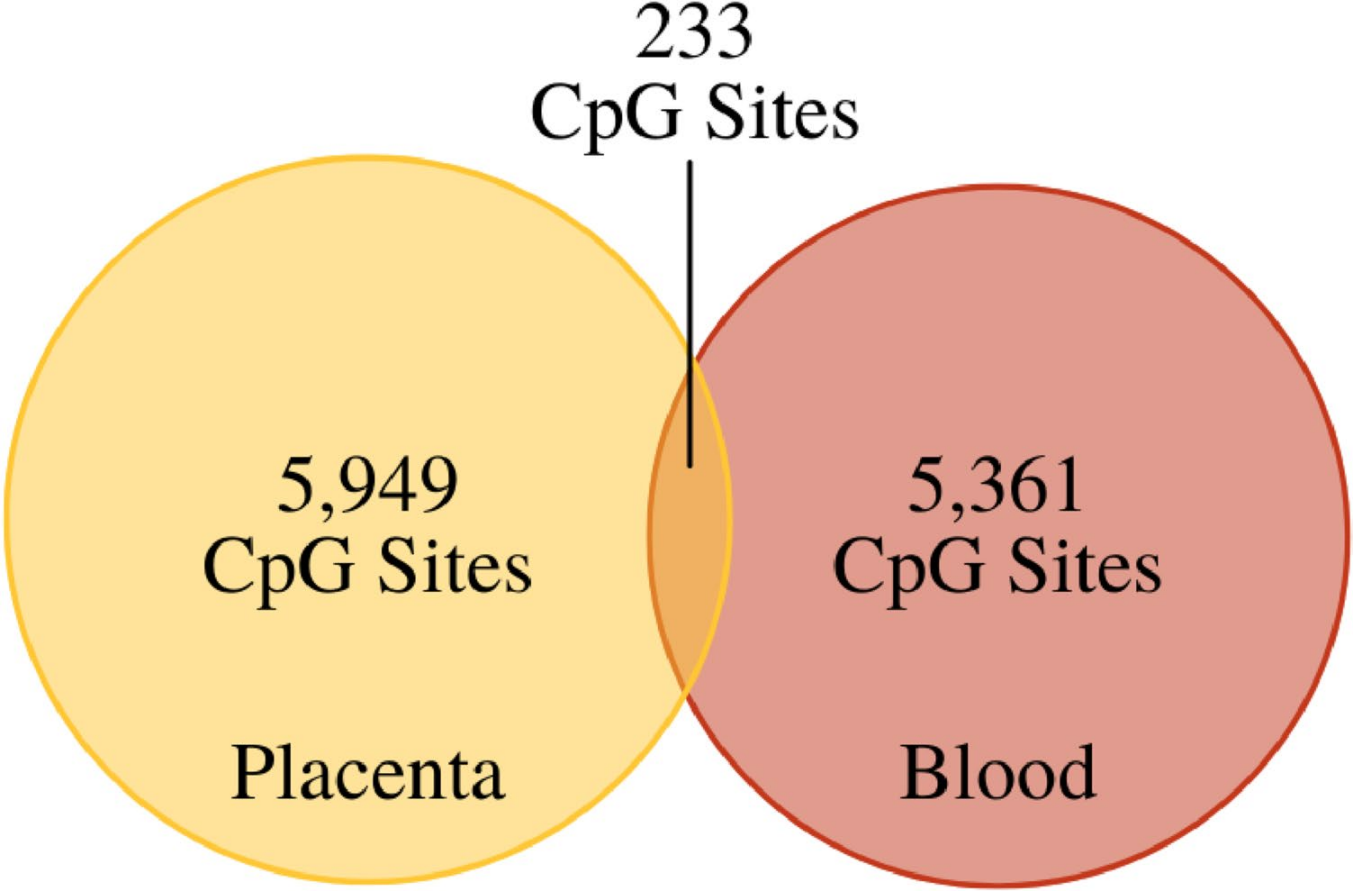
Comparison of CpG site methylation associated with sex between the placenta and blood. A total of 11,543 CpG sites displayed sexually dimorphic patterns in methylation: 5949 of these sites were found only in the placenta, and 5361 were found only in the blood sample. Of all sites, 233 (2.0%) were differentially methylated in both tissues

We investigated sexually dimorphic patterns of CpG methylation in the placenta and blood, controlling for cell-type heterogeneity. Our analysis identified 11,543 significantly differentially methylated autosomal CpG loci based on sex (Fig. 1). Among these, 51.5% (6182) were placenta-specific, and 46.4% (5594) were blood-specific. It is noteworthy that we observed minimal overlap between the placenta and blood, with only 2% of the CpG loci (233 CpG annotated to 165 genes) showing differential methylation in both tissues (Fig. 2). Additional file 1: Table S1 in the supplemental material provides the list of all significant CpG loci identified.

**Fig. 3:**
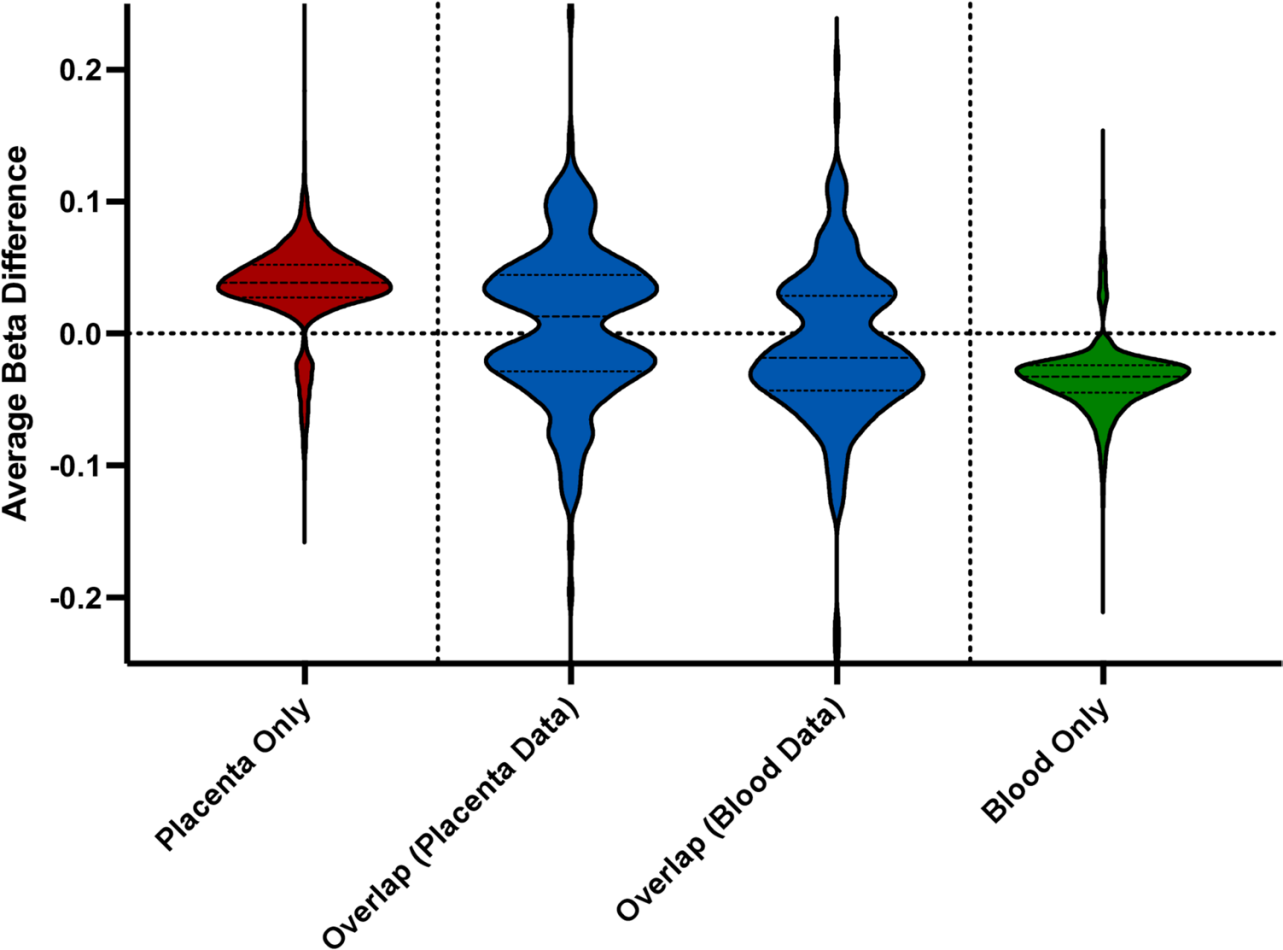
Distribution of the average beta value differences between males and females for all sexually dimorphic CpG sites. Beta differences greater than 0 represent hypermethylation in males, and beta differences below 0 represent hypermethylation in females. Note the hypermethylation in males in placenta-only CpG sites and hypermethylation in females in blood-only CpG sites

In the placenta-specific findings, among the most significantly differentially methylated CpG loci, males had *higher* average methylation levels as compared to females (Fig. 3, Table 2). The three most significantly differentially methylated annotated CpG loci in male and female placentas were found to be cg11532947 (logFC = 0.96, FDR 5.25E − 117), cg07355069 (logFC = 1.03, FDR 4.71E − 95), and cg25801066 (logFC = 0.76, FDR 4.89E − 79), all of which are located in the regulatory regions of genes. The CpG site cg11532947, located in the 3′UTR region of the NGFI-A binding protein 1 (*NAB1*) gene, demonstrated higher average methylation levels in males than in females (84% vs 72%, respectively). Similarly, the CpG site cg07355069, located in the 5′UTR region of the 3-hydroxy-3-methylglutaryl-CoA synthase 1 (*HMGCS1*) gene, demonstrated higher average methylation levels in males than in females (92% vs 85%, respectively). Finally, the CpG site cg25801066, located in the 3′UTR region of the 3 Calmodulin 1 (*CALM1*) gene, demonstrated significantly higher average methylation levels in males than in females (76% vs 65%, respectively) (Table 3).

**Table 2:** A summary of sexually dimorphic CpG sites in the blood, placenta, or both tissues considered together. Results are presented as (A) total CpGs found, including tissue-specific and overlapping; (B) unique, tissue-specific CpGs; and (C) overlapping CpGs in each tissue. For overlapping CpGs, the loci are identical between blood and placenta tissue, but methylation levels vary

	**Blood**		**Placenta**		**All**	
**(A) Total CpGs**	5594		6182		11,543	
*Annotated to known genes*	3840	68.6%	3751	60.7%	7426	64.3%
*Hypermeth. in males*	362	6.5%	5507	89.1%		
**(B) Tissue-specific CpGs**	5361	95.8%	5949	96.2%	11,310	98.0%
*Annotated to known genes*	3675	68.6%	3586	60.3%	7261	64.2%
*Hypermeth. in males*	277	5.2%	5389	90.6%	5666	50.1%
**(C) CpGs common to both tissues**	233	4.2%	233	3.8%	233	2.0%
*Annotated to known genes*	165	70.8%	165	70.8%	165	70.8%
*Hypermeth. in males*	85	36.5%	118	50.6%

**Table 3:** Top 25 most sexually dimorphic CpG sites found in the (A) blood only, (B) placenta only, (C) planceta and blood (blood methylation levels shown). The annotated gene is provided where known

CpG	Gene	Chrom	CpG location	*P*-value	FDR	logFC^a^	Male Avg. beta	Female Avg. beta	Sex Avg. beta difference
**A) Placenta specific**									
cg11532947	*NAB1*	2	3′UTR	5.89E − 122	5.25E − 117	0.966	0.840	0.728	0.113
cg07355069	*HMGCS1*	5	5′UTR	7.04E − 100	4.71E − 95	1.035	0.926	0.859	0.067
cg25801066	*CALM1*	14	3′UTR	1.10E − 83	4.89E − 79	0.765	0.762	0.656	0.106
cg18090212	*APP*	21	3′UTR	9.04E − 72	3.30E − 67	0.467	0.864	0.824	0.041
cg24276949	*RIC8B*	12	5′UTR	1.48E − 69	5.16E − 65	0.649	0.784	0.702	0.081
cg09102486		5		1.27E − 55	3.09E − 51	0.678	0.608	0.499	0.109
cg17264064	*ACAA2*	18		1.67E − 52	3.72E − 48	0.785	0.682	0.559	0.123
cg00761634	*CHTF8*	16	3′UTR	6.38E − 51	1.35E − 46	0.854	0.930	0.869	0.061
cg19473894	*CCDC121*	2	TSS1500	1.00E − 50	2.02E − 46	0.680	0.656	0.556	0.100
cg11730618	*RAB7A*	3	TSS1500	1.82E − 50	3.56E − 46	0.813	0.813	0.712	0.101
cg03289961	*HADHB*	2	ExonBnd	1.72E − 49	3.28E − 45	0.896	0.595	0.452	0.143
cg01440070	*SLC6A10P*	16	3′UTR	8.10E − 47	1.39E − 42	0.818	0.748	0.636	0.112
cg17968869	*SPON1*	11	TSS1500	1.90E − 43	2.99E − 39	0.694	0.863	0.793	0.069
cg08721112		1		2.90E − 43	4.40E − 39	0.339	0.531	0.471	0.060
cg07285048		11	TSS1500	4.33E − 43	6.44E − 39	0.674	0.913	0.864	0.049
cg25667734	*TERF1*	8	TSS1500	4.92E − 41	7.06E − 37	0.683	0.883	0.823	0.060
cg11634496		11		1.09E − 39	1.49E − 35	− 0.927	0.136	0.222	− 0.087
cg02343988		11	TSS200	1.61E − 39	2.16E − 35	0.439	0.653	0.580	0.073
cg09779044	*CKAP5*	11	5′UTR	1.63E − 38	2.08E − 34	0.639	0.834	0.765	0.068
cg18905668	*GTF2H1*	11	3′UTR	1.21E − 37	1.43E − 33	0.418	0.608	0.537	0.072
cg15604132	*ZNF300*	5	TSS1500	1.72E − 37	2.00E − 33	1.118	0.569	0.395	0.174
cg27149150	*CAMTA1*	1	TSS1500	2.60E − 37	2.99E − 33	0.828	0.471	0.343	0.128
cg22846149	*HMGB1L1*	20		2.88E − 37	3.26E − 33	0.591	0.626	0.530	0.096
cg09914736	*CAMTA1*	1		3.14E − 36	3.50E − 32	0.767	0.699	0.588	0.111
cg22936497		12		3.39E − 36	3.73E − 32	− 0.348	0.466	0.525	− 0.060
**B) Blood specific**									
cg04946709	*LOC644649*	16	TSS1500	2.23E − 89	7.75E − 85	0.752	0.793	0.703	0.091
cg07585845		3	TSS200	1.25E − 81	3.81E − 77	0.764	0.725	0.614	0.111
cg11284736	*HDGFRP3*	15		1.10E − 80	3.10E − 76	0.686	0.828	0.753	0.075
cg25742246		1	3′UTR	1.04E − 58	2.12E − 54	− 0.749	0.050	0.080	− 0.029
cg16021537	*RBMS1*	2	1stExon	1.14E − 53	1.94E − 49	− 0.911	0.056	0.094	− 0.038
cg03253995	*EIF4A1*	17	5′UTR	3.20E − 52	5.32E − 48	− 0.450	0.436	0.512	− 0.076
cg19311244		4	3′UTR	7.68E − 47	1.08E − 42	− 0.601	0.518	0.618	− 0.099
cg11092486		6		1.85E − 45	2.56E − 41	− 0.806	0.371	0.500	− 0.129
cg17561891	*C7orf23*	7	TSS1500	1.91E − 45	2.58E − 41	− 0.441	0.166	0.210	− 0.044
cg05812269	*RIOK3*	18	5′UTR	4.70E − 45	6.24E − 41	− 0.460	0.115	0.149	− 0.034
cg24920126	*PPP1R3G*	6	1stExon	1.46E − 44	1.91E − 40	− 0.994	0.384	0.548	− 0.164
cg13045294	*LTBP4*	19	5′UTR	2.88E − 44	3.69E − 40	− 0.611	0.438	0.538	− 0.100
cg07607752	*ZPBP2*	17	TSS200	2.84E − 43	3.51E − 39	− 1.142	0.111	0.203	− 0.092
cg09639931	*ZPBP2*	17	TSS200	3.07E − 43	3.74E − 39	− 0.951	0.152	0.249	− 0.098
cg08528995	*SLC35D3*	6		4.28E − 43	5.12E − 39	− 1.033	0.705	0.823	− 0.118
cg05330360	*ZPBP2*	17	TSS1500	1.54E − 42	1.76E − 38	− 0.508	0.327	0.404	− 0.078
cg09044186	*APOA5*	11		1.85E − 42	2.08E − 38	− 1.263	0.776	0.883	− 0.107
cg03405128		4	3′UTR	4.47E − 42	4.95E − 38	− 0.503	0.304	0.382	− 0.077
cg21784396	*PRRT4*	7		4.02E − 41	4.32E − 37	− 0.963	0.035	0.061	− 0.027
cg25294504		20	3′UTR	7.05E − 41	7.47E − 37	− 0.644	0.673	0.758	− 0.085
cg26897297	*MLNR*	13	3′UTR	2.48E − 40	2.55E − 36	− 0.483	0.578	0.659	− 0.080
cg11574745	*PPP1R3G*	6	1stExon	2.84E − 40	2.84E − 36	− 0.516	0.446	0.532	− 0.086
cg20808136		15		2.84E − 40	2.84E − 36	0.492	0.853	0.810	0.043
cg07004386		14		3.85E − 40	3.80E − 36	− 0.650	0.275	0.369	− 0.095
cg08319905	*PPFIA3*	19	ExonBnd	8.20E − 40	7.99E − 36	− 0.313	0.432	0.487	− 0.054
**C) Placenta and blood overlap**									
cg26919182	*PPP1R12B*	1	5′UTR	1.68E − 253	1.23E − 247	− 3.004	0.525	0.894	− 0.368
cg12691488		1		6.27E − 221	2.29E − 215	2.606	0.264	0.059	0.206
cg15228509		1		4.91E − 212	1.20E − 206	− 1.684	0.399	0.670	− 0.270
cg06513015	*ERV3-1*	7	5′UTR	1.69E − 194	3.09E − 189	− 1.781	0.625	0.846	− 0.221
cg00148935	*RFTN1*	3	3′UTR	2.10E − 176	3.07E − 171	− 1.606	0.531	0.769	− 0.239
cg09516963	*DYRK2*	12	TSS1500	3.03E − 171	3.69E − 166	− 2.519	0.136	0.423	− 0.286
cg11643285	*RFTN1*	3	3′UTR	1.13E − 167	1.18E − 162	− 1.500	0.776	0.904	− 0.127
cg03626220	*HYDIN*	16		2.66E − 144	2.43E − 139	0.703	0.680	0.567	0.114
cg26355737	*TFDP1*	13		4.90E − 127	3.98E − 122	0.997	0.888	0.801	0.087
cg03226871		2	5′UTR	3.17E − 125	2.31E − 120	0.685	0.605	0.488	0.118
cg02716779	*DNM1*	9	3′UTR	3.08E − 124	2.04E − 119	− 0.799	0.557	0.681	− 0.125
cg02325951	*FOXN3*	14	1stExon	1.12E − 119	6.79E − 115	1.026	0.678	0.510	0.169
cg12607525	*UBTF*	17		2.30E − 119	1.29E − 114	0.589	0.591	0.490	0.101
cg20262915	*NAB1*	2	1stExon	7.43E − 116	3.88E − 111	0.690	0.543	0.428	0.115
cg20299935		17		8.80E − 115	4.29E − 110	− 0.948	0.622	0.756	− 0.133
cg19765154	*NAB1*	2	1stExon	4.63E − 114	2.11E − 109	0.878	0.806	0.698	0.108
cg23719534		15	3′UTR	1.40E − 110	6.03E − 106	− 1.591	0.827	0.918	− 0.092
cg03618918		1		1.02E − 105	4.15E − 101	0.784	0.847	0.767	0.080
cg17238319	*RFTN1*	3		2.33E − 105	8.97E − 101	− 0.965	0.682	0.799	− 0.117
cg17232883		11		8.96E − 98	3.27E − 93	− 0.802	0.078	0.125	− 0.047
cg07852945	*TLE1*	9	TSS1500	8.89E − 85	2.95E − 80	− 1.019	0.054	0.101	− 0.047
cg17612569	*GABPA*	21	5′UTR	4.35E − 82	1.38E − 77	1.100	0.078	0.039	0.040
cg02989351	*YWHAQ*	2	1stExon	7.40E − 81	2.16E − 76	− 0.644	0.094	0.138	− 0.043
cg03218192	*AP2B1*	17	1stExon	4.57E − 79	1.24E − 74	− 0.681	0.276	0.379	− 0.104
cg09696045		15	TSS1500	9.09E − 78	2.37E − 73	− 0.412	0.387	0.458	− 0.071

^a^logFC is the effect size corresponding to a “beta” value in a regression, i.e., the average change in *y* for a 1 unit change in *x*. In this paper, this would correspond to a change in *M* value when going from sex = 0 (female) to 1 (male)

In the blood-specific findings, among the most significantly differentially methylated CpG loci, males had *lower* average methylation levels as compared to females (Fig. 3, Table 2). The three most significantly differentially methylated annotated CpG loci in male and female blood were found to be cg04946709 (logFC = 0.75, FDR 7.75E − 85), cg11284736 (logFC = 0.68, FDR 3.10E − 76), and cg16021537 (logFC =  − 0.91, FDR 1.94E − 49), all of which are in the regulatory regions of genes. The CpG site cg04946709, located in the TSS1500 region of the apolipoprotein O pseudogene 5 (*LOC644649*) gene, demonstrated higher average methylation levels in males than in females (79% vs 70%, respectively). Similarly, the CpG site cg11284736, located in the hepatoma-derived growth factor-related protein 3 (*HDGFRP3*) gene, demonstrated higher average methylation levels in males than in females (82% vs 75%, respectively). Finally, the CpG site cg16021537, located in the 1st exon of the RNA-binding motif single-stranded interacting protein 1 (*RBMS1*) gene, demonstrated lower average methylation levels in males than in females (5% vs 9%, respectively) (Table 3).

In both tissues, the CpG loci with the most significant differential methylation levels in were cg26919182 (logFC =  − 3.00, FDR 1.23E − 247) located in the 5′UTR region of the protein phosphatase 1 regulatory subunit 12B (*PPP1R12B*) gene on chromosome 1, with an average methylation of 52% in males and 89% in females; cg06513015 (logFC =  − 1.78, FDR 3.09E − 189) located in the 5′UTR region of the 3-endogenous retrovirus group 3 member 1 envelope (*ERV3-1*) gene on chromosome 7, with an average methylation of 62% in males and 84% in females; and cg00148935 (logFC =  − 1.60, FDR 3.07E − 171) located in the 3′UTR region of the raftlin lipid raft linker 1 (*RFTN1*) gene on chromosome 3, with an average methylation of 52% in males and 76% in females.

Enrichment analysis revealed six Gene Ontology categories significantly associated with the placenta-specific CpG loci (Table 4), with the most significant being keratinocyte differentiation (FDR 1.43E − 05) and keratinization (FDR 1.66E − 05). No significant associations were observed for the blood-specific CpG loci or the CpG loci found in both tissues.

**Table 4:** Gene Ontology enrichment results for CpG loci identified as significant in placenta specific analysis

	**Ontology**	**Term**	***N***	**DE**	**P.DE**	**FDR**
GO:0030216	BP	Keratinocyte differentiation	136	8	6.26E − 10	1.43E − 05
GO:0031424	BP	Keratinization	55	6	1.46E − 09	1.66E − 05
GO:0009913	BP	Epidermal cell differentiation	198	8	1.41E − 08	0.0001
GO:0008544	BP	Epidermis development	320	9	3.34E − 08	0.0002
GO:0043588	BP	Skin development	260	8	1.40E − 07	0.0006
GO:0001533	CC	Cornified envelope	44	4	2.23E − 06	0.0085

*BP* biological process, *CC* cellular component, *N* number of genes in the GO or KEGG term, *DE* number of genes that are differentially methylated, *P.DE p* value for over-representation of the GO or KEGG term

## Discussion

Children who are born prematurely exhibit sexually dimorphic patterns in health outcomes during and after the neonatal period [1, 4–7]. However, the biological mechanisms that cause the differences in health outcomes between males and females are not well-studied. In this study, we aimed to identify and compare patterns of CpG methylation in the placenta and blood that could be responsible for the sexually dimorphic health outcomes in children. Our findings revealed notable differences in CpG methylation patterns between males and females in both tissues. In males, there was a general trend of hypomethylation in blood and hypermethylation in the placenta. The study also found that there was limited overlap in the CpG sites that were sexually dimorphic between the placenta and blood, indicating that genes that are methylated in a dimorphic manner have specific tissue responses.

Our study found significant differences in CpG loci methylation levels in placenta-specific genes, with the most significant changes observed in *NAB1*, *HMGCS1*, and *CALM1* genes. *NAB1* is involved in regulating gene expression and neuronal differentiation. One study found that *NAB1* was differentially methylated in the placenta and cord blood samples based on infant sex [12]. *HMGCS1* is critical for cholesterol and ketone body metabolism and has been previously identified to be methylated in the placenta and cord blood in a sexually dimorphic manner [12]. Importantly, *HMGCS1* has been implicated in placental function. For example, a study found that *HMGCS1* is associated with vascular dysfunction of the placenta [36]. Regarding *CALM1*, this gene encodes calmodulin, a calcium-binding protein involved in cell signaling. *CALM1* has been implicated in regulating uterine contractions and preeclampsia [37]. A term placenta study, however, did not find DNA methylation of *CALM1* to be sexually dimorphic [38].

Similarly in the blood-specific findings, we identified significant differential methylation in several CpG loci, with the most statistically significant ones being annotated to the *LOC644649* and *RBMS1* genes. *LOC644649* encodes for apolipoprotein O, a protein involved in lipid metabolism and cardiovascular disease [39], and has been showing sexually dimorphic DNA methylation patterns in cord blood samples [12]. *RBMS1* is required for radial migration, polarization, and differentiation of neuronal progenitors to neurons in the neocortex development [40] but has not shown a sexually dimorphic pattern in a blood DNA methylation analysis [41]. Our study identified several genes with significantly differentially methylated CpG sites in both placenta and blood, with potential implications for sex dimorphism in child health outcomes. The *PPP1R12B*, *ERV3-1*, and *RFTN1* genes were the most significantly differentially methylated in both tissues. Of interest, *PPP1R12B* encodes for the protein phosphatase 1 regulatory subunit, which has been implicated in a range of biological processes, including cell motility and contractility. *RFTN1* encodes for raftlin, a protein involved in lipid raft signaling. Like our findings, another study found sexually dimorphic DNA methylation patterns in the placenta and cord blood for CpG loci in *PPP1R12B* and *RFTN1* [12].

Our study supports the previously reported existence of sexually dimorphic DNA methylation patterns in fetal tissues, including the placenta and umbilical cord blood, which are associated with sex-specific differences in the gene expression and fetal development [12, 42]. Specifically, we found that 893 differentially methylated CpG loci in the placenta tissue and 1382 CpG loci in the blood were in common with the findings of Bozack et al. [12]. Additionally, we identified 3750 CpG loci for blood that were in common with Solomon et al.’s study [42]. The list of common CpG loci is provided in Additional file 1: Table S1. Bozack et al. [12] examined the associations between infant sex and DNA methylation across the umbilical cord blood, artery, and placenta samples. The study found significant sex-based differences in DNA methylation patterns in all three tissue types, with males having lower average methylation levels than females at specific CpG sites. Solomon et al. performed a meta-analysis of the association of sex and cord blood DNA methylation at over 450,000 CpG sites in 8438 newborns from 17 cohorts participating in the Pregnancy And Childhood Epigenetics (PACE) Consortium [42].

Interestingly, methylation patterns of the sexually dimorphic CpG sites differed between tissues. In the placenta, roughly 90% of all sexually dimorphic CpG sites were hypermethylated in males; the reverse was true for blood, with approximately 95% of sexually dimorphic CpG sites hypermethylated in females. This unique reversal of methylation level was expected and has been described previously but is not well understood [10–12, 15]. However, we observed minimal overlap (only 2% of the CpG loci) between the differentially methylated CpG sites in the placenta and blood, suggesting the existence of tissue-specific epigenetic regulation mechanisms in the context of sexual dimorphism. CpG methylation is known to play a role in cell differentiation and tissue-specific function and was therefore expected to vary between the placenta and blood to an extent [12]. One possible explanation for the tissue specificity observed here is that both the placenta and blood tissues have unique structures and functions for males and females, controlled, at least in part, by CpG methylation. In the placenta, there are sex differences in size, shape, vasculature, and gene expression [43]. In the blood, there are known differences in the metabolomic profiles [44], rheologic properties (viscosity, red blood cell aggregation, and oxygen delivery index) [45], and immune cell concentrations from childhood through adulthood [46]. The genes (and related CpG sites) that control the sex-specific structure and function of these tissues are expected to differ between tissues, potentially explaining the minimal amount of overlapping sexually dimorphic CpGs observed here.

## Conclusions

Several factors should be considered when interpreting the results of this study. First, the ELGAN cohort comprises children who were all born extremely preterm (born before 28 weeks of gestation), potentially limiting the generalizability of our results. Because inflammation is a risk factor for preterm birth, the specific CpG methylation levels identified here may not be generalizable for tissues collected from term infants. Second, we compared DNA methylation patterns between two tissues (placenta and blood) to identify similarities in patterns associated with sex differences. However, although we adjusted for cell type, sex differences in DNA methylation between placenta and blood could be affected by cell types so the common CpGs are of interest. DNA methylation is tissue-specific, and differences in methylation of individual CpG sites are probably less important than sexually dimorphic methylation patterns in the blood and placenta. However, these results only capture two perinatal tissues and may not be representative of the comprehensive methylome across the body. Lastly, this analysis did not include transcriptional data; thus, we could not determine which methylation findings are associated with gene expression function. This study provides valuable and unique information about the sexual dimorphism of the placental and blood methylomes. Future studies should address these limitations to continue elucidating the biological mechanisms surrounding sexual dimorphism that are linked to differential health and disease risk among children.

## Methods

### The ELGAN cohort

This study included data from the Extremely Low Gestational Age Newborn (ELGAN) cohort, which was enrolled in a prospective study designed to examine the risk of structural and neurologic disorders in extremely preterm children [47]. Between 2002 and 2004, women delivering before 28 weeks of gestation were asked to enroll in the study from five states (North Carolina, Massachusetts, Michigan, Illinois, Connecticut) across 14 participating institutions. Institutional Review Board approval was obtained at each site. Overall, 1249 mothers of 1506 infants were enrolled, but only 415 participants had placenta specimens and 390 had neonatal blood analyzed for DNA methylation. Of the participants with DNA methylation data, 358 had both placenta and peripheral blood biospecimens analyzed for DNA methylation that allowed us to complete the analysis for this manuscript (detailed further below). Previous studies have shown no systemic difference between the complete ELGAN cohort and the subset of the samples with DNA methylation [19, 48].

### Placenta tissue and neonatal blood collection

Placenta tissue collection within the ELGAN cohort has been described in detail elsewhere [15, 49, 50]. Briefly, at delivery, the placentas were collected and placed in a sterile basin and transferred to a sampling room where they were biopsied. A sample (< 1 g) of fetal-derived placental tissue was biopsied by pulling back the chorion and amnion. Sterile 2-mL cryovials with the samples were submerged in liquid nitrogen and stored in a − 80 °C freezer. Using blood spot filter paper cards (Schleicher & Schuell 903, GE Healthcare, Chicago, IL), neonatal blood was collected at postnatal day 1 (range, 1–3 days) and stored at − 70 °C in sealed bags with desiccant until processing.

### DNA extraction and methylation assessment

The placental samples were sliced into ~ 0.2-g segments with a sterile dermal curette. The segments were then washed in 1 × PBS (Fisher Scientific, Waltham, MA) to reduce any potential blood contamination. Following washing, samples were immediately snap-frozen in homogenization tubes and placed back on dry ice. The final processing step involved homogenizing the tissue segments using a sterile stainless-steel bead (Qiagen, Germantown, MD) in RLT + lysis buffer (Qiagen) with the TissueLyserII instrument (Qiagen). This clarifies the samples through spinning to remove the cellular debris and the bead. Homogenized samples were stored at − 80 °C until nucleic acid extraction.

Blood spots were collected on filter paper (Schleicher & Schuell 903, GE Healthcare, Chicago, IL) and stored at − 70 °C in sealed bags with desiccant until processing. To extract DNA from the dried blood spots, 3-mm diameter spots were punched from the filter paper, lysed in Proteinase K solution from the EZ1 DNA Investigator kit (Qiagen, Germantown, MD) and shaken in a thermomixer. The resulting supernatant was processed using the Qiagen EZ1 Advanced instrument according to the manufacturer’s protocol. The quantity of DNA was assessed using a DropSense 96 Spectrophotometer (Trinean, Pleasanton, CA), with a minimum of 20 ng DNA considered acceptable for methylation analysis.

The ALLPrep DNA/RNA/miRNA Universal Kit (Qiagen) was used to isolate DNA sequences > 18 nucleotides long. Bisulfite conversion was then performed using the EZ DNA Methylation Kit (Zymo Research, Irvine, CA). Methylation status was quantified utilizing the Illumina Infinium MethylationEPIC BeadChip (Illumina, San Diego, CA), which can evaluate methylation at more than 850,000 CpG sites across the genome.

### Methylation data quality assessment and quality control

All the analyses described here were conducted in the R software. In the processing and normalization of the DNA methylation data, we evaluated sex mismatches using the minfi (v1.36.0) package [51], removed probes that failed quality control (*p* value > 0.01), removed outliers, and assessed and controlled for batch effects. For the placental methylation data, these steps removed 4 samples for sex mismatches and removed zero samples based on the sample-based filter. For the dried blood spot methylation data, these steps removed 18 samples for sex mismatches and 4 samples based on the sample filter. These steps resulted in a total of 358 samples for the current analysis. Additionally, 1597 (of 850,000) CpG loci from the placental methylation data and 79,458 (of 850,000) loci from the dried blood spot methylation data were removed through the probe-based filter. The ShinyMethyl (v1.26.0) package was used simultaneously with the above steps to visually verify the data [52]. The data were then normalized utilizing the normal-exponential out-of-band (*noob*) correction method and functional normalization [53]. Batch effects were identified using principal components analysis in combination with visualizing data using plate position, chip, array, and date covariates. Plate position was determined to potentially be inducing batch effects in the placenta and dried blood spot methylation data. Batch effects were removed utilizing the ComBat function within the sva (v3.38.0) package [54, 55]. Following batch effect correction, final *β* values were generated and then converted to *M* values ([log_2_(*β*/(1 − *β*)]), as these are more statistically valid for subsequent analysis [56].

### Cell type estimation

To control for cell-type effects on DNA methylation patterns, cell-type proportions were empirically estimated in both the placenta and dried blood spot data. Cell type estimation for the placenta methylation data was conducted utilizing the *planet* package (v0.99.4) [57–60]. In order to obtain a comprehensive understanding of the cell-type variation observed in the second trimester placenta samples used in this study (ranging from 161 to 191 gestational days or 23–28 weeks), we calculated the average of the first and third trimester libraries. This enabled us to capture the full range of cell types present in the samples more effectively. For the blood data, cell type estimation was conducted utilizing the FlowSorted.Blood.EPIC package (v1.8.0), specifically utilizing the cord blood-derived reference library [61, 62].

### Statistical analysis

Epigenome wide-associated studies (EWAS) were conducted separately for the placental and blood spot DNA methylation datasets. For both placental and blood spot methylations, we evaluated the association between CpG methylation and sex while excluding cross-reactive probes and probes annotated to X and Y chromosomes. Cross-reactive probes were previously generated by Chen et al. using an approach to identify array probes potentially generating spurious signals due to co-hybridization to alternate sequences homologous to intended targets [63]. Sex chromosomes were excluded from this analysis for three reasons: (1) sex chromosomes have unique patterns of gene regulation compared to autosomes. The X chromosome, for example, is subject to X-inactivation in females, which results in the silencing of one of the two X chromosomes, while the Y chromosome has a limited number of genes. These unique features of the sex chromosomes make it difficult to compare DNA methylation patterns between males and females; (2) the sex chromosomes are present in different numbers in males and females. Females have two X chromosomes, while males have one X and one Y chromosome. This difference in the number of sex chromosomes could potentially affect DNA methylation patterns and lead to false-positive or false-negative associations; and (3) the exclusion of sex chromosomes from sex dimorphism analysis allows for better statistical power and more reliable results.

Models were fit for each CpG locus using the CpG *M* value as the response variable and sex as the main predictor using robust linear regression with the *limma* package (v3.46.0) [64]. All models were adjusted for cell type heterogeneity using the PCA-derived variables (described previously). Moderated test statistics were calculated using an empirical Bayes method to shrink probe-wise sample variance towards a common value and control for test-statistic inflation using the ebayes function in *limma*. *p* values were considered significant after using the Bonferroni correction method with an *α* value of 0.05.

### Pathway enrichment analysis

To investigate the biological pathways associated with sexually dimorphic CpG sites, enrichment analyses were performed using *missMethyl* package in R [65]. The analysis included all statistically significant sexually dimorphic CpG sites found (1) only in the placenta, (2) only in blood, and (3) in both placenta and blood tissues.

### Supplementary Information

**Additional file 1: Table S1.** Provides a list of all significant CpG loci identified in the analysis presented in this manuscript.

## Data Availability

All data analyzed during this study are included in this published article and its supplemental information files and publicly available at the NCBI Gene Expression Omnibus GSE167885.
